# Histological response to radiotherapy is an early event in myxoid liposarcoma

**DOI:** 10.1007/s00428-023-03615-5

**Published:** 2023-08-12

**Authors:** Suk Wai Lam, Tulio M. Silva, Jolanda Traast-Kooistra, Inge Briaire-de Bruijn, Brendy van den Akker, Pauline A. C. Bakker, Jules Lansu, Rick L. M. Haas, Judith V. M. G. Bovée

**Affiliations:** 1 Department of Pathology, Leiden University Medical Center, Leiden, the Netherlands; 2 Department of Pathology, Vall d´Hebron University Hospital, Barcelona, Spain; 3 Department of Radiotherapy, Leiden University Medical Center, Leiden, the Netherlands; 4 Sarcoma Unit, Department of Radiotherapy, the Netherlands Cancer Institute, Amsterdam, the Netherlands

**Keywords:** Myxoid liposarcoma, Radiotherapy, Personalized medicine

## Abstract

Compared to other sarcomas, myxoid liposarcoma (MLS) is exceptionally sensitive to radiation therapy, but the underlying mechanism remains unknown. The objective was to assess the tissue-based changes in MLS during and after neoadjuvant radiotherapy in 26 patients of the DOREMY trial. Morphological assessment was performed on biopsies pre-treatment, after 8 fractions, 16 factions, and after surgical resection and included percentage of viable tumor cells, hyalinization, necrosis, and fatty maturation. Furthermore, immunohistochemistry was performed for apoptosis (cleaved caspase-3), anti-apoptosis (Bcl-2), activity of mTOR signaling (phospho-S6), hypoxia (CAIX), proliferation (Ki67), inflammation (CD45 and CD68), and microvessel density (CD34 Chalkley count). A pronounced reduction in vital tumor cells was observed early with a drop to 32.5% (median) tumor cells (IQR 10–93.8%) after 8 fractions. This decreased further to 10% (IQR 5–30%) after 16 fractions and 7.5% (IQR 5–15%) in the surgical specimen. All but one patient had an excellent response with < 50% remaining tumor cells. Inversely, treatment response was mainly observed as hyalinization and less often as fatty maturation. Additionally, a decrease of inflammatory cells was noticed especially during the first eight fractions. Microvessel density remained stable over time. Immunohistochemical markers for apoptosis, anti-apoptosis, activity of mTOR signaling, proliferation, and hypoxia did not show any marked changes within the remaining tumor cells during and after radiotherapy. As a modest dose of neoadjuvant radiotherapy induces profound tissue changes in MLS, mainly during the first 8 fractions, current findings might suggest that in a carefully selected patient population further deintensification of radiotherapy might be explored.

**Supplementary information:**

The online version contains supplementary material available at 10.1007/s00428-023-03615-5.

## Introduction

Myxoid liposarcoma (MLS) is a malignant lipomatous tumor, typically arising within the deep soft tissues of the extremities, especially of the thigh. Morphologically, these tumors are composed of uniform round to oval cells and lipoblasts, arranged in a myxoid background with a characteristic capillary-sized branching vasculature. High-grade MLS, formerly known as round cell liposarcoma, has a component (> 5% of the tumor) of overlapping round cells with a higher nuclear grade with loss of the characteristic myxoid and vascular background [1]. These tumors are associated with a higher metastatic rate and worse prognosis [2, 3]. On molecular level, the majority of MLS harbor a *FUS::DDIT3* fusion [4], or less often *FUS* is substituted by *EWSR1* [5]. It is postulated that the chimeric fusion product functions as an aberrant transcription factor stimulating proliferation and blocking adipocytic differentiation [6].

Among different soft tissue sarcomas, MLS is one of most radiosensitive [7]. While in a preoperative setting, a standard total dose of 50 Gy in 1.8–2 Gy is usually given for soft tissue sarcoma [8], successful deintensification of preoperative radiotherapy dose in MLS was recently reported in the DOREMY trial [9]. In this study, a total dose of 36 Gy in a once-daily 2-Gy fractions was given, followed by definitive surgical resection, which did not compromise the oncological outcome. Currently, the underlying mechanism of this particular radiosensitivity is unknown. As MLS harbor a capillary rich vascular network, a characteristic morphological feature, we hypothesized that radiotherapy might exerts its function by damaging this specific capillary network. Another possibility might be related to the induction of differentiation, since maturation of adipocytes is a distinct response pattern seen in MLS, originally described after treatment with trabectedin [10] and subsequently also reported in patients treated with radiation therapy [11]. It was shown that this phenomenon was the result of activation of transcriptional program promoting terminal differentiation of adipocytes [12]. Furthermore, since the immunomodulatory effect of radiotherapy on the immune micro-environment has been increasingly recognized [13], this might be of crucial importance in determining the success in therapy [14].

This study aims at characterizing tissue-based changes through morphological and immunohistochemical analysis. The unique sample set of consecutive biopsies taken prior and during neoadjuvant radiation in the DOREMY trial enables the assessment of tissue-based changes occurring during and after neoadjuvant radiotherapy to ultimately decipher why MLSs are particularly radiosensitive.

## Material and methods

### Samples

We evaluated 26 patients from the Netherlands with translocation-confirmed primary MLS from the prospective, single-arm, phase 2 DOREMY clinical trial [9]. Treatment consisted of neoadjuvant radiotherapy to 36 Gy in once-daily 2-Gy fractions, followed by definitive surgical resection. Evaluation was performed at four different timepoints: biopsy pre-treatment, biopsy after eight fractions, biopsy after sixteen fractions, and after definitive surgical resection. Clinical characteristics are summarized in Table 1.

**Table 1:** Clinical and pathological characteristics

DOREMY number	Age	Gender	Location	Tumor size (cm)	Fusion	Histological grade	Clear surgical margin
5	37	M	Proximal lower limb	11.5	*FUS::DDIT3*	Low	Yes
7	59	M	Other	7	*FUS::DDIT3*	Low	Yes
8	39	F	Distal lower limb	20.3	*FUS::DDIT3*	Low	Yes
10	48	F	Proximal lower limb	8.8	*FUS::DDIT3*	Low	Yes
11	30	M	Proximal lower limb	13	*FUS::DDIT3*	Low	Yes
14	40	M	Distal lower limb	9.9	*FUS::DDIT3*	Low	Yes
15	50	F	Proximal lower limb	10.2	*FUS::DDIT3*	Low	N/A
16	48	M	Proximal lower limb	6.4	*FUS::DDIT3*	Low	Yes
17	61	M	Proximal lower limb	17.9	*FUS::DDIT3*	High	Yes
19	36	F	Distal lower limb	6.3	*FUS::DDIT3*	Low	Yes
30	34	F	Proximal lower limb	13	*FUS::DDIT3*	Low	Yes
31	66	M	Proximal lower limb	7.4	*FUS::DDIT3*	Low	Yes
33	43	M	Proximal lower limb	8	*FUS::DDIT3*	High	Yes
34	25	F	Distal lower limb	8	*FUS::DDIT3*	High	Yes
35	44	F	Distal lower limb	11	*FUS::DDIT3*	Low	Yes
49	49	F	Proximal lower limb	16.7	*FUS::DDIT3*	Low	Yes
55	33	F	Proximal lower limb	9.4	*EWSR1::DDIT3*	High	Yes
58	47	M	Proximal lower limb	9.1	*FUS::DDIT3*	Low	Yes
65	45	M	Proximal lower limb	15.2	*FUS::DDIT3*	Low	Yes
71	52	F	Proximal lower limb	4.6	*FUS::DDIT3*	Low	Yes
73	20	M	Proximal lower limb	12.9	*FUS::DDIT3*	Low	Yes
78	36	M	Proximal lower limb	5.1	*FUS::DDIT3*	N/A	Yes
81	40	M	Proximal lower limb	18.8	*FUS::DDIT3*	Low	Yes
84	25	F	Proximal lower limb	6.5	*EWSR1::DDIT3*	Low	Yes
85	59	M	Proximal lower limb	19.7	*FUS::DDIT3*	Low	Yes
92	53	M	Proximal lower limb	17.9	*FUS::DDIT3*	Low	Yes

### Morphological evaluation

Morphological features were assessed on H&E, and the percentage of viable tumoral cells, hyalinization/fibrosis, tumoral necrosis, and fatty maturation was estimated on the biopsies and all available sections of the resection specimen according to the modified EORTC STBSG scoring system for response [15, 16]. Percentage added up to a sum of 100%. All slides were scored by two independent readers. Descriptive statistics were used to analyze the pathological outcomes and were reported as mean ± standard deviation or median (IQR) where appropriate. Statistical analysis was performed using the Friedman test with an additional post hoc analysis.

### Immunohistochemistry

Tissue microarrays (TMAs) were constructed as described previously [17]. From each specimen, a pathologist selected representative tumor areas for tissue cores with a diameter of 1.5 mm. To outweigh intratumoral heterogeneity, three cores from each sample were taken whenever possible.

Immunohistochemistry for anti-apoptosis (Bcl2), activity of mTOR signaling (phospho-S6) and hypoxia (CAIX), apoptosis (cleaved caspase-3), proliferation (Ki67), inflammation (CD45 and CD68), and microvessel density (CD34 Chalkley count) was performed on TMAs using standard lab protocols. In short, after deparaffinization, microwave antigen retrieval was performed in Tris–EDTA (pH 9.0) or citrate (pH 6.0). Details of antibodies are summarized in supplementary Table 1. Overnight incubation of the antibody followed, whereafter detection with power vision Poly-HRP (immunologic, the Netherlands) and visualization with a DAB + substrate chromogen system followed. Lastly, slides were counterstained with hematoxylin, dehydrated, and mounted.

Markers for anti-apoptosis (Bcl2), activity of mTOR signaling (phospho-S6), and hypoxia (CAIX) were scored semi-quantitatively by two independent readers, blinded for clinical data and time sequence, according to intensity (weak, moderate, or strong) and percentage of positive cells (0, 0%; 1 + , 1–25%; 2 + , 25–50%; 3 + , 50–75%; or 4 + , 75–100%), as previously described [18]. In case of disagreement among the two initial readers, a third reader served as an adjudicator. Markers for apoptosis (percentage of cleaved caspase-3-positive nuclei), proliferation (percentage of Ki67-positive nuclei), inflammation (CD45-positive cells/mm^2^ and CD68-positive cells/mm^2^), and microvessel density (CD34 Chalkley count) [19] were analyzed automatically with digital software for image analysis (QuPath).

## Results

### Morphological evaluation

Morphological re-evaluation of the biopsy prior to neoadjuvant treatment was possible in seventeen patients. In eighteen patients, the biopsy after eight fractions and sixteen fractions could be assessed, while in all patients (*n* = 26), the surgical specimen was available for further analysis. In twelve patients, materials of all four timepoints were available for morphological analysis.

In the pre-treatment biopsies, three cases were classified as high-grade MLS, with a round cell component varying between 25 and 45%. In thirteen cases, the diagnostic biopsy was completely composed of vital tumor cells. In the remaining four biopsies, the percentage of viable tumor cells ranged from 50 to 95%. In these cases, mature adipocytes were observed (range 5–10%). In addition, focal necrosis was present in one case (2% of biopsy), and two cases had hyalinization prior to treatment (5% and 40% of biopsy).

**Fig. 1:**
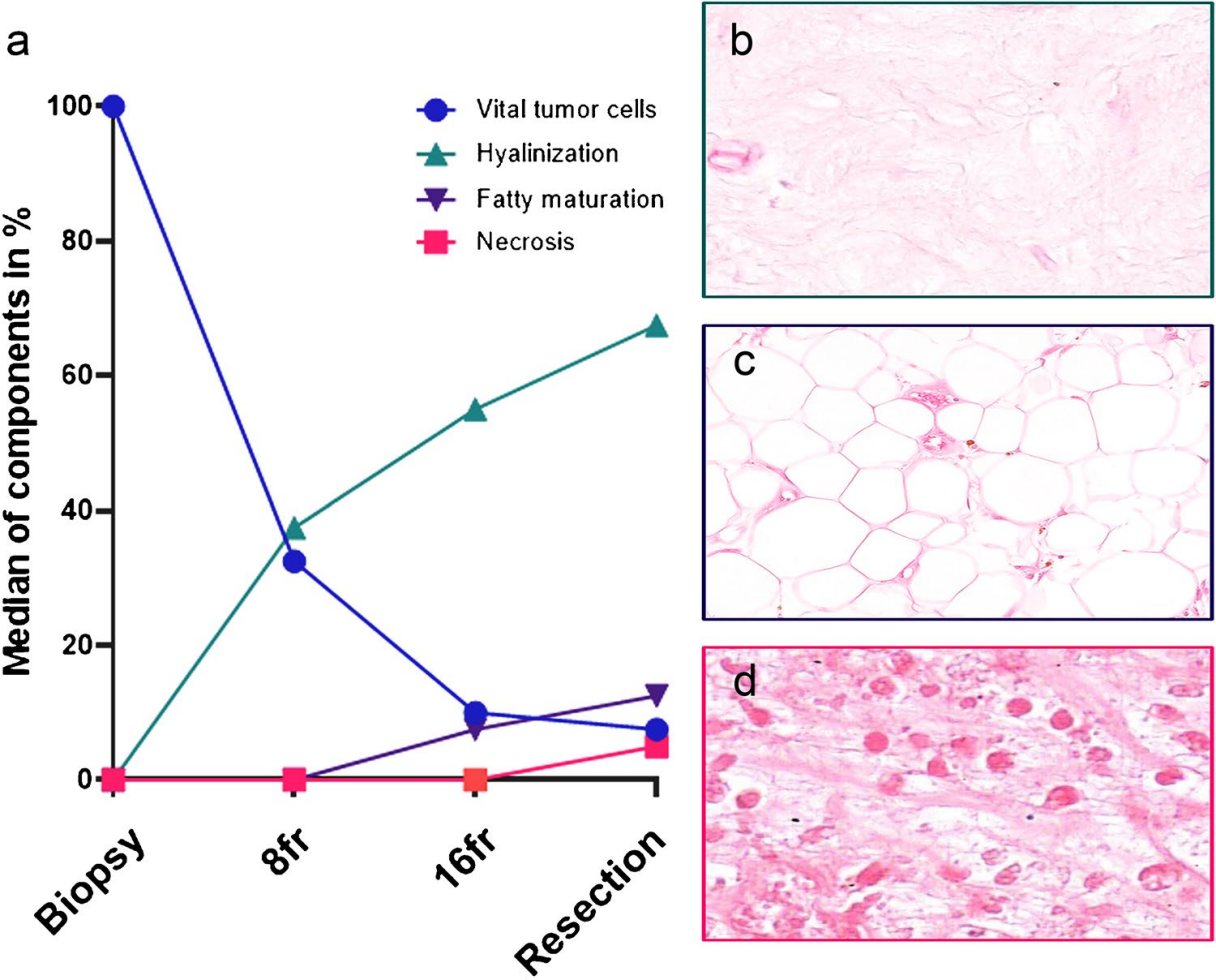
Morphological evaluation revealed a decrease in vital tumor cells during treatment. Reduction of vital tumor cells was most pronounced early, during the first eight fractions (**a**). Inversely, the histological effect of radiation treatment was observed predominantly as hyalinization (**b**) followed by fatty maturation (**c**), while tumor necrosis (**d**) was less prominent

Morphological evaluation revealed a decrease in vital tumor cells during treatment to a median of 32.5% (IQR 10–94%) after eight fractions and declined to 10% (IQR 5–30%) and 7.5% (IQR 5–15%) after sixteen fractions and in the surgical specimen, respectively. Notably, the reduction of vital tumor cells was most pronounced early, before fraction eight (Fig. 1a). Most patients (25/26) had a good response, defined as ≥ 50% treatment effect in the surgical specimen [9]. In eighteen cases (18/26, 69%), the percentage of viable tumor cells was 10% or less. Differences in remaining vital tumor cells were not observed between high-grade MLS and low-grade MLS. None of the cases showed a complete pathological response with no residual tumor cells. Inversely, increased hyalinization was the most pronounced histological change induced by the irradiation (Fig. 1a, b). The percentage increased to a median of 38% after eight fractions (IQR 6–60%), whereafter it climbed up to 55% (IQR 24–80%) after sixteen fractions and 67.5% (IQR 29–80%) in the resection specimen. Fatty maturation was less pronounced and comprised only a small fraction of the response in the on-treatment biopsies with a median of 0% (IQR 0–13%) after eight fractions and a median of 7.5% (IQR 0–34%) after sixteen fractions. Furthermore, it was rather a late event compared to hyalinization with a median of 13% (5–31%) in the resections specimen (Fig. 1a, c). Tumor necrosis was rarely observed in the on-treatment biopsies (median 0% (IQR 0–4%) and 0% (IQR 0–9%)) but was slightly more often present in the surgical specimen with a median of 5% (IQR 0–11%) (Fig. 1a, d).

**Fig. 2:**
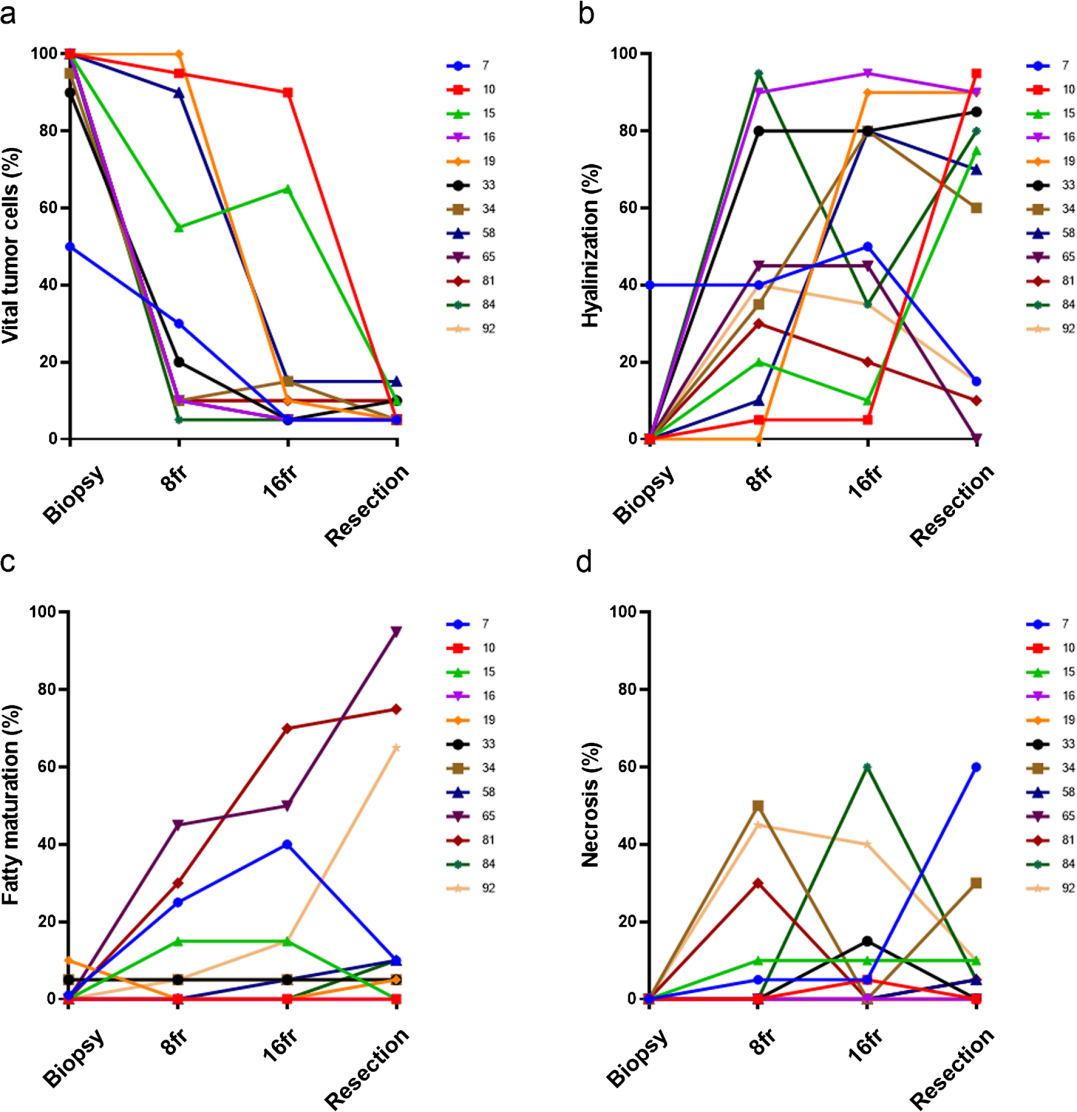
Morphological response evaluation of paired samples in individual patients. Most patients (8/12) were early responders with < 50% vital tumor cells readily after eight fractions, while two patients had an extensive response after sixteen fractions. The two remaining patients were late responders (**a**). Hyalinization was an early event and comprised the largest response component in most patients (**b**). In the remaining patients, fatty maturation was most outspoken with a gradual increase during treatment (**c**). Necrosis was variably present in individual patients (**d**)

When assessing the morphological response in the patients of which the specimens for all timepoints were available (i.e., paired samples), similar trends were observed regarding the reduction of vital tumor cells. Already after eight fractions, the majority of patients (8/12, 67%) had less than 50% of vital tumor cells, thus extensive treatment response as defined in the DOREMY trial [9], although not statistically significant (Dunn’s multiple comparisons test, *p* = 0.07). In the remaining four cases, two patients had extensive response after sixteen fractions, while the other two patients were late responders with eventually extensive response in the resection specimen (Fig. 2a). Significant differences in vital tumor cells were found between the pre-treatment biopsy and after sixteen fractions (Dunn’s multiple comparisons test, *p* < 0.001), as well as between the pre-treatment biopsy and the resection (Dunn’s multiple comparisons test, *p* < 0.001). Hyalinization appeared to be an early event and the most pronounced histological change in the fast majority (Fig. 2b). When comparing the pre-treatment biopsy to the on-treatment biopsies and the resection, significant changes were found for each comparison (Dunn’s multiple comparisons test, *p* = 0.01, *p* = 0.002, and *p* = 0.01, respectively). In contrast, fatty maturation was a relatively late event and appeared to be the largest component of response in only three patients, while in the remaining cases, it only comprised a minor part of the histological change (Fig. 2c). Treatment effect was less often observed as necrosis and was also heterogeneous among the different patients (Fig. 2d).

**Fig. 3:**
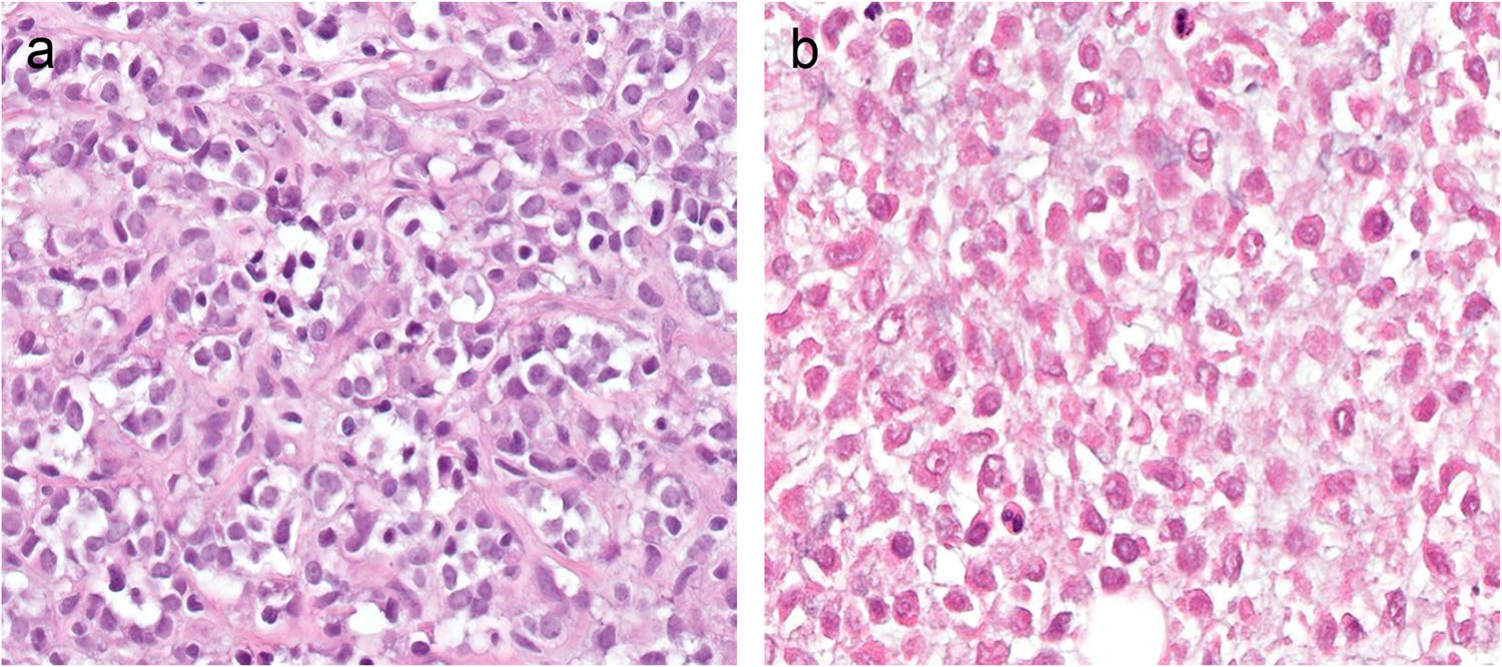
Biopsy pre-treatment shows high-grade round cell component (**a**) that became completely necrotic after eight fractions (**b**)

**Fig. 4:**
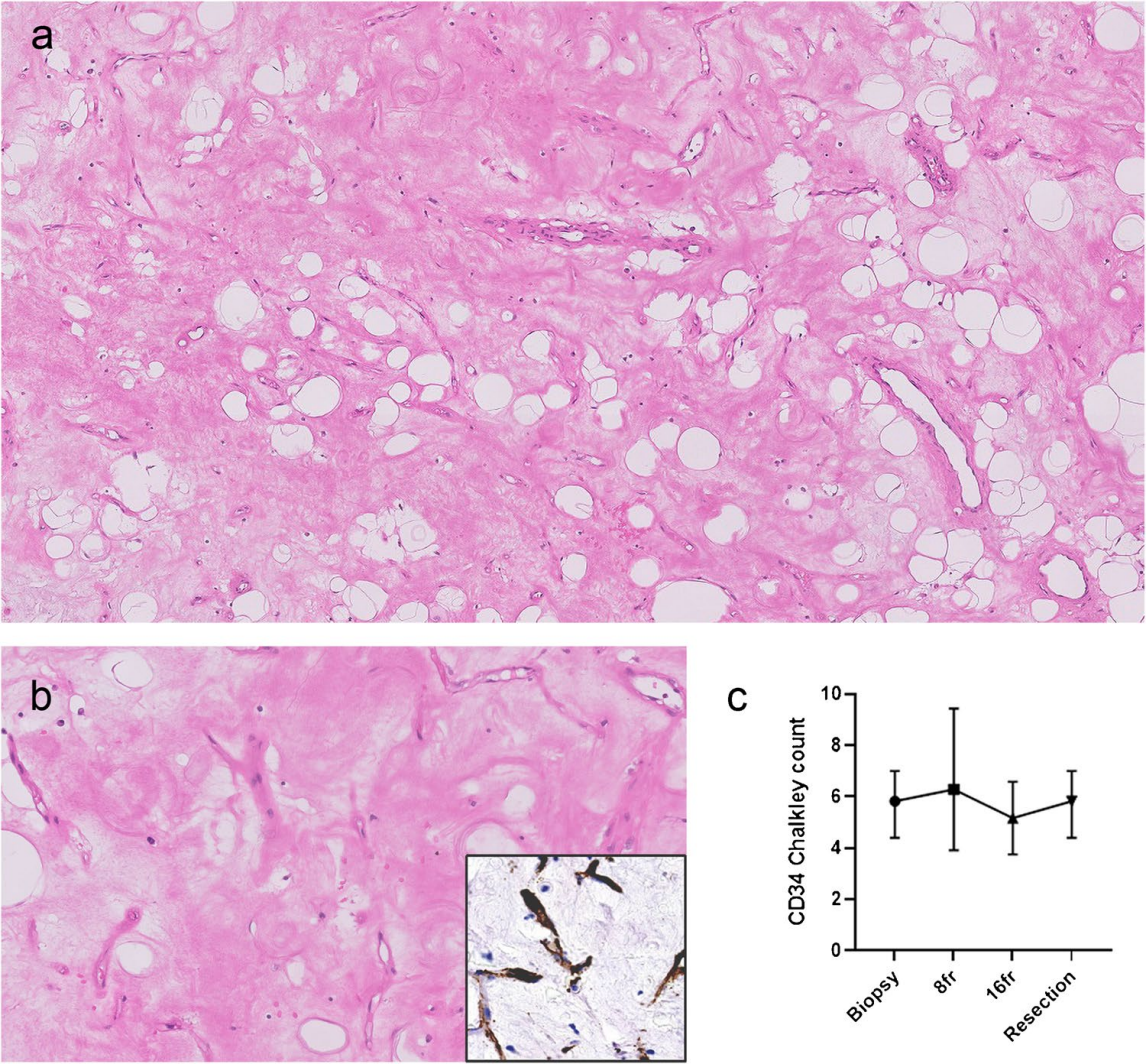
The characteristic branching capillary network remains present in the resection specimen after neoadjuvant radiation, low-power image (**a**) and high-power image (**b**) which are highlighted using CD34 immunohistochemistry (insert). Mean vascular density using CD34 Chalkley count remained stable over time (**c**). Symbols represent median, and error bars represent IQR

In one high-grade MLS, the round cell component became fully necrotic after eight fractions (Fig. 3). In the two remaining high-grade MLS cases, all on-treatment biopsies showed only conventional low-grade MLS morphology. The high-grade component was not visible in the treatment biopsies. Interestingly, the arborizing capillary network remained present in the areas with hyalinization (Fig. 4a, b).

### Immunohistochemical evaluation

**Fig. 5:**
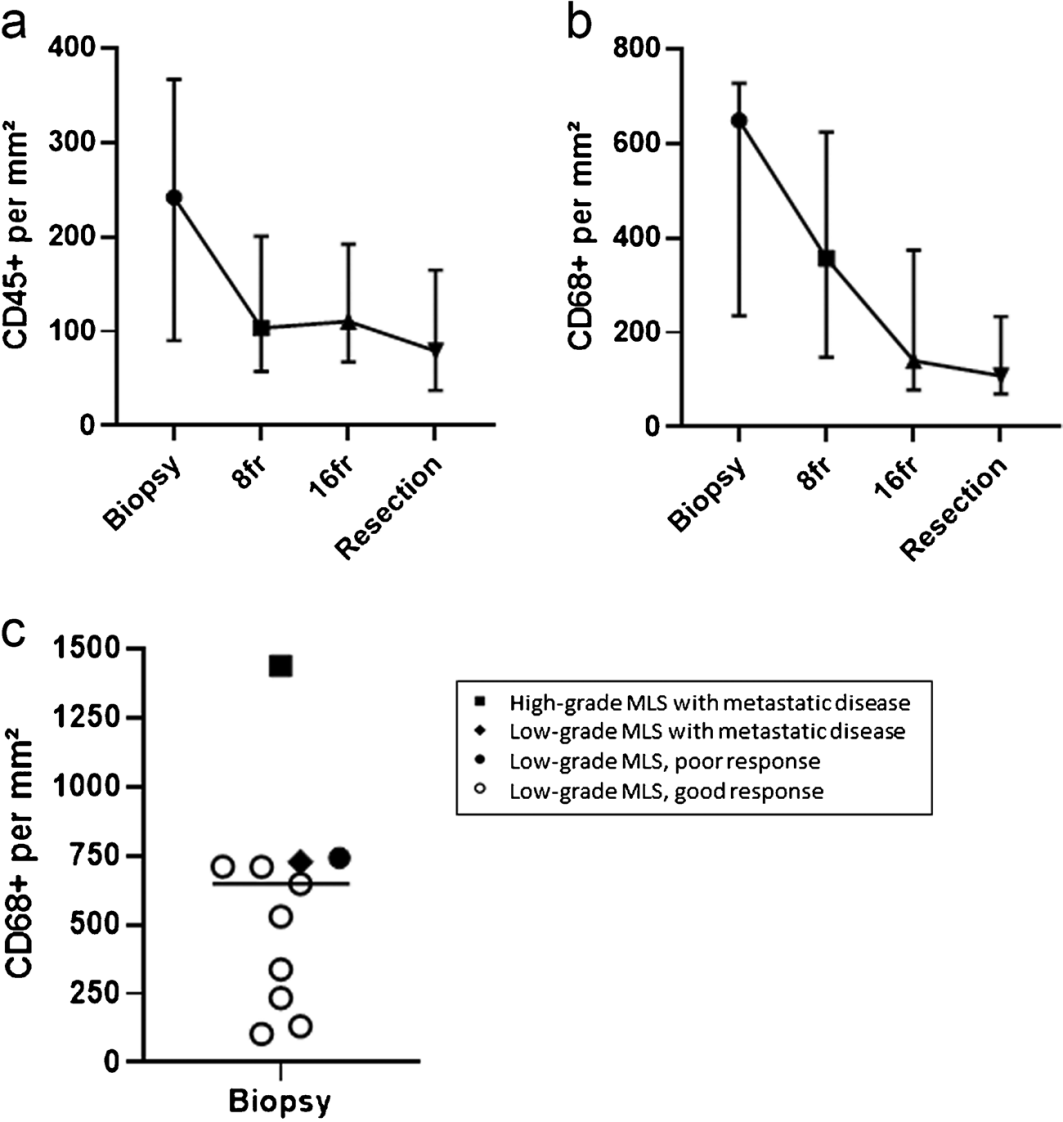
Immunohistochemistry shows a decline of CD45 + immune cells during the first eight fractions (**a**), whereas the amount of macrophages (CD68) further drops during treatment. Symbols represent median, and error bars represent IQR (**b**). Amount of CD68 + cells in biopsy before treatment (**c**). Patient with high-grade morphology, metastatic disease, and poor response tends to have higher levels of macrophages. Each symbol represents one myxoid liposarcoma patient

In concordance with the morphological observation, the microvessel density determined by CD34 positivity remained stable over time with a median Chalkley count of 5.8, 6.2, 5.2, and 5.8 in the consecutive specimens (Fig. 4c), indicating that the arborizing capillary network was unaffected by the radiation. The number of tumor infiltrating immune cells as determined by CD45 and CD68 counts showed an overall decrease during the first eight fractions, however not statistically significant. The CD68-positive cells (macrophages) (median of 648/mm^2^ (IQR 235–727/mm^2^)) outnumbered the CD45-positive cells (median of 242/mm^2^ (IQR 58–366/mm^2^)) in the tumor micro-environment before treatment. Compared to the pre-treatment biopsy, a decline in CD45 + cells was observed after eight fractions, where after it remained stable (Fig. 5a). The amount of CD68 + macrophages decreased to a median of 357/mm^2^ (IQR 148–623/mm^2^) after eight fractions and 141/mm^2^ (IQR 79–374/mm^2^) after sixteen fractions. The number of macrophages was the lowest in the resection specimens with a median of 109/mm^2^ (IQR 71–234/mm^2^) (Fig. 5b). Notably, the two patients with distant metastasis had high numbers of macrophages (1439/mm^2^ and 727/mm^2^) in the diagnostic biopsy. One of these patients was diagnosed with high-grade MLS and had a worse histological response with 25% viable tumor cells compared to the other patients (median of 8% remaining viable tumor cells). Remarkably, the patient with the poorest response (75% residual tumor) also had high levels of CD68 + macrophages (Fig. 5c).

Overall, the proliferation rate (Ki67) was already low prior to treatment with a median of 2% (IQR 0.8–3%) and remained stable over time within the remaining viable tumor cells, with no differences between high-grade and low-grade MLS. Similarly, immunohistochemical markers for apoptosis (cleaved caspase-3), hypoxia (CAIX), and anti-apoptosis (Bcl-2) were stably low before and after treatment, while the activity of mTOR signaling (phospho-S6) was high (median IHC score of 6 (5–6)) prior to treatment and slightly declined after radiation therapy (median IHC score of 4 (IQR 3.3–5)), though not statistically significant (Supplementary Fig. 1).

### Follow-up

Thus far, none of the patients had a local recurrence after a median follow-up time of 36 months (range: 3.1–87.5 months). Two patients developed distant metastasis. One had pleural metastasis, and the other had metastasis to the omentum.

## Discussion

Histopathological changes in MLS were assessed in a unique sample set of tissues obtained before, during, and after a moderate dose of neoadjuvant radiotherapy, allowing the observation of radiotherapy-related changes over time. Profound morphological tissue changes and alterations in tumor micro-environment were observed, with most effect already observed during the first eight fractions.

Radiation induced a large reduction of vital tumor cells that was inversely correlated to increased hyalinization and to a lesser extent adipocytic maturation and necrosis, as was also described by others [20, 21]. In general, radiation exerts its effect by damaging DNA and thereby inducing cell cycle arrest, senescence, apoptosis, DNA damage repair [22, 23], and by damaging capillaries and small vessels [23]. In line with other studies, radiation did not affect the vascularity in MLS [20, 21]. In addition, no marked changes in markers related to apoptosis, activity of mTOR, autophagy, and hypoxia were found, indicating that its radiosensitivity might be caused by other mechanisms. Notably, MLS showed a low proliferation level, even in the high-grade tumors. This might be explained by the high levels of cyclin-dependent kinase inhibitors found in MLS cells [24], counteracting growth promoting activity, as it was postulated that the aberrant FUS-DDIT3 transcription factor would result in stimulation of proliferation, while inhibiting adipocytic differentiation. Interestingly, radiation of MLS cells enhanced expression of p21, causing cell cycle arrest [21], leading to differentiation and maturation. This might, at least partially, explain the response observed in myxoid liposarcoma visible as lipoma-like areas.

Remarkably, most tissue changes were already observed after the first eight fractions. Since none of the patients developed a local recurrence, it is questionable whether further reduction of radiotherapy, potentially resulting in lower wound complications and late toxic effects, would affect local control. Correlation between radiological and pathological response in MLS is mostly assessed using the surgical specimen and imaging after neoadjuvant treatment [21, 25, 26]. Recently, our group demonstrated that MRI following sixteen fractions could be contributive in the prediction of the pathological treatment response [27], suggesting that patients with an excellent response might be omitted from the last two fractions. MRI after eight fractions was not of predictive value [27]. This indicates that correlation between the pathological and radiological response might not be optimal and that radiological changes might fall behind. Further research is needed to decipher whether further reduction of radiotherapy is possible, without compromising the outcome.

Alterations in the micro-environment were observed, with a major reduction in CD45 + immune cells and CD68 + macrophages during the first eight fractions and a further decrease of CD68 + macrophages over time. Literature of the immune environment after radiotherapy in MLS is sparse. Snow et al. described a mixed response of CD68 positivity after radiation in a diverse cohort of different liposarcomas, which depended on the method of analysis [28]. Others demonstrated that the inflammatory micro-environment in MLS was capable of producing different cytokines, chemokines, and growth factors and that the chimeric protein FUS-DDIT3 was involved in the activation of the inflammatory cascade [29]. Similar to our findings, mice transplanted with MLS had lower infiltration of CD68 + macrophages after trabectedin treatment and a showed reduction of other key inflammatory mediators (CCL2, CXCL8, IL-6, and VEGF) in vitro and in vivo [29]. Further studies should elucidate whether similar effects occur after radiation in myxoid liposarcoma.

Notably, an abundant presence of CD68 + macrophages in the pre-treatment biopsy was predominantly seen in patients that developed distant metastasis in high-grade MLS and patients with a poorer response after radiation. In line with these findings, in a multivariate analysis, macrophage infiltration predicted a poor prognosis in MLS [30]. In addition, Minopoli et al. showed that high-grade MLS possesses a T-cell-poor and macrophage-rich phenotype, associated with a poor outcome, while low-grade MLS had a discrete amount of T cells and low levels of M2-like macrophages. Furthermore, co-culturing of MLS cells and macrophages drove a M2-like phenotype, increasing the invasive capability in MLS cells [31]. Altogether, these results suggest an important role of tissue-associated macrophages in promoting immune-related activities for tumor progression.

With respect to the limitations of this study, we used TMAs for immunohistochemical analysis. Although TMAs are proven to be representative for the whole tumor [32], we noticed frequent detachment of biopsy cores probably due to the myxoid matrix, hampering parts of the immunohistochemical analysis and loss of tissue cores. Furthermore, intermittent assessment of pathological response on biopsies during treatment could have led to some degree of sampling error, particularly in large tumors with a heterogeneous response. Also, as MLSs are rare tumors, our patient cohort was relatively small hampering extensive statistical analysis. Lastly, as we were not able to find any dominant pathways of radiogenic cell loss, analysis of the expression of proteins such as ATM, ATR, and CHK1 that control the G2 checkpoint might be interesting to evaluate, as inhibition of these promote mitotic catastrophe, which has been acknowledged as a main form of cell death induced by radiation [33].

## Conclusion

A moderate dose of neoadjuvant radiotherapy induces profound tissue changes in myxoid liposarcoma, with the therapeutical effect predominantly occurring during the first eight fractions. High levels of macrophages prior to treatment were associated with high-grade MLS, metastasis, and poor response to therapy, and the number of macrophages decreased during treatment. Future research is warranted to investigate if further deintensification is possible in a carefully selected patient population. By integrating intermitted assessment of radiological and pathological response together with elements of the immune micro-environment, future decision-making may shift away from mainstay treatment towards personalization of treatment strategies including radiation dose.

### Supplementary information

Below is the link to the electronic supplementary material.

Supplementary file1 (DOCX 13 KB)

Supplementary file2 (TIF 17939 KB)

## Declarations

All patients included in the DOREMY clinical trial provided written informed consent before enrollment. The trial protocol and all amendments to it were approved by the institutional review board or research ethics committee of each of the 9 participating sarcoma centers. All samples are coded according to the Dutch code of proper secondary use of human material as accorded by the Dutch society of pathology (Federa).

## References

[1] WHO Classification of Tumours Editorial Board: Soft Tissue and bone tumours. Fifth ed. Lyon (France): International Agency Research on Cancer; 2020.

[2] Haniball J, Sumathi VP, Kindblom LG, Abudu A, Carter SR, Tillman RM (2011). Prognostic factors and metastatic patterns in primary myxoid/round-cell liposarcoma. Sarcoma.

[3] Fiore M, Grosso F, Lo Vullo S, Pennacchioli E, Stacchiotti S, Ferrari A (2007). Myxoid/round cell and pleomorphic liposarcomas: prognostic factors and survival in a series of patients treated at a single institution. Cancer.

[4] Crozat A, Aman P, Mandahl N, Ron D (1993). Fusion of CHOP to a novel RNA-binding protein in human myxoid liposarcoma. Nature.

[5] Bode-Lesniewska B, Frigerio S, Exner U, Abdou MT, Moch H, Zimmermann DR (2007). Relevance of translocation type in myxoid liposarcoma and identification of a novel EWSR1-DDIT3 fusion. Genes Chromosomes Cancer.

[6] Adelmant G, Gilbert JD, Freytag SO (1998). Human translocation liposarcoma-CCAAT/enhancer binding protein (C/EBP) homologous protein (TLS-CHOP) oncoprotein prevents adipocyte differentiation by directly interfering with C/EBPbeta function. J Biol Chem.

[7] Callegaro D, Miceli R, Bonvalot S, Ferguson P, Strauss DC, Levy A (2018). Impact of perioperative chemotherapy and radiotherapy in patients with primary extremity soft tissue sarcoma: retrospective analysis across major histological subtypes and major reference centres. Eur J Cancer.

[8] Gronchi A, Miah AB, Dei Tos AP, Abecassis N, Bajpai J, Bauer S (2021). Soft tissue and visceral sarcomas: ESMO-EURACAN-GENTURIS Clinical Practice Guidelines for diagnosis, treatment and follow-up(☆). Ann Oncol.

[9] Lansu J, Bovée J, Braam P, van Boven H, Flucke U, Bonenkamp JJ (2021). Dose reduction of preoperative radiotherapy in myxoid liposarcoma: a nonrandomized controlled trial. JAMA Oncol.

[10] Forni C, Minuzzo M, Virdis E, Tamborini E, Simone M, Tavecchio M (2009). Trabectedin (ET-743) promotes differentiation in myxoid liposarcoma tumors. Mol Cancer Ther.

[11] Wang WL, Katz D, Araujo DM, Ravi V, Ludwig JA, Trent JC (2012). Extensive adipocytic maturation can be seen in myxoid liposarcomas treated with neoadjuvant doxorubicin and ifosfamide and pre-operative radiation therapy. Clin Sarcoma Res.

[12] Di Giandomenico S, Frapolli R, Bello E, Uboldi S, Licandro SA, Marchini S (2014). Mode of action of trabectedin in myxoid liposarcomas. Oncogene.

[13] Colton M, Cheadle EJ, Honeychurch J, Illidge TM (2020). Reprogramming the tumour microenvironment by radiotherapy: implications for radiotherapy and immunotherapy combinations. Radiat Oncol.

[14] Barker HE, Paget JT, Khan AA, Harrington KJ (2015). The tumour microenvironment after radiotherapy: mechanisms of resistance and recurrence. Nat Rev Cancer.

[15] Wardelmann E, Haas RL, Bovée JV, Terrier P, Lazar A, Messiou C (2016). Evaluation of response after neoadjuvant treatment in soft tissue sarcomas; the European Organization for Research and Treatment of Cancer-Soft Tissue and Bone Sarcoma Group (EORTC-STBSG) recommendations for pathological examination and reporting. Eur J Cancer.

[16] Schaefer IM, Hornick JL, Barysauskas CM, Raut CP, Patel SA, Royce TJ (2017). Histologic appearance after preoperative radiation therapy for soft tissue sarcoma: Assessment of the European Organization for Research and Treatment of Cancer-Soft Tissue and Bone Sarcoma Group Response Score. Int J Radiat Oncol Biol Phys.

[17] Cleven AH, Hocker S, Briaire-de Bruijn I, Szuhai K, Cleton-Jansen AM, Bovee JVMG (2015). Mutation analysis of H3F3A and H3F3B as a diagnostic tool for giant cell tumor of bone and chondroblastoma. Am J Surg Pathol.

[18] Lam SW, Cleven AH, Briaire-de Bruijn IH, Bovee JVMG (2019). Utility of FOS and FOSB immunohistochemistry in osteoid osteoma and osteoblastoma. Lab Invest.

[19] Vermeulen PB, Gasparini G, Fox SB, Toi M, Martin L, McCulloch P (1996). Quantification of angiogenesis in solid human tumours: an international consensus on the methodology and criteria of evaluation. Eur J Cancer.

[20] Salduz A, Alpan B, Valiyev N, Özmen E, İribaş A, Ağaoğlu F (2017). Neoadjuvant radiotherapy for myxoid liposarcomas: oncologic outcomes and histopathologic correlations. Acta Orthop Traumatol Turc.

[21] Engström K, Bergh P, Cederlund CG, Hultborn R, Willen H, Aman P (2007). Irradiation of myxoid/round cell liposarcoma induces volume reduction and lipoma-like morphology. Acta Oncol.

[22] Baskar R, Dai J, Wenlong N, Yeo R, Yeoh KW (2014). Biological response of cancer cells to radiation treatment. Front Mol Biosci.

[23] Wang JS, Wang HJ, Qian HL (2018). Biological effects of radiation on cancer cells. Mil Med Res.

[24] Olofsson A, Willén H, Göransson M, Engström K, Meis-Kindblom JM, Stenman G (2004). Abnormal expression of cell cycle regulators in FUS-CHOP carrying liposarcomas. Int J Oncol.

[25] Wortman JR, Tirumani SH, Tirumani H, Shinagare AB, Jagannathan JP, Hornick JL (2016). Neoadjuvant radiation in primary extremity liposarcoma: correlation of MRI features with histopathology. Eur Radiol.

[26] Roberge D, Skamene T, Nahal A, Turcotte RE, Powell T, Freeman C (2010). Radiological and pathological response following pre-operative radiotherapy for soft-tissue sarcoma. Radiother Oncol.

[27] Lansu J, Braam PM, van Werkhoven E, Scholten AN, Schrage Y, van Houdt WJ (2021). A moderate dose of preoperative radiotherapy may improve resectability in myxoid liposarcoma. Eur J Surg Oncol.

[28] Snow H, Mitchell C, Hendry S, McKinley M, Byrne D, Ngan S (2021). Characterising the immune microenvironment in liposarcoma, its impact on prognosis and the impact of radiotherapy. J Surg Oncol.

[29] Germano G, Frapolli R, Simone M, Tavecchio M, Erba E, Pesce S (2010). Antitumor and anti-inflammatory effects of trabectedin on human myxoid liposarcoma cells. Cancer Res.

[30] Nabeshima A, Matsumoto Y, Fukushi J, Iura K, Matsunobu T, Endo M (2015). Tumour-associated macrophages correlate with poor prognosis in myxoid liposarcoma and promote cell motility and invasion via the HB-EGF-EGFR-PI3K/Akt pathways. Br J Cancer.

[31] Minopoli M, Sarno S, Cannella L, Tafuto S, Scognamiglio G, Gallo M (2021). Crosstalk between macrophages and myxoid liposarcoma cells increases spreading and invasiveness of tumor cells. Cancers (Basel).

[32] Sauter G (2010). Representativity of TMA studies. Methods Mol Biol.

[33] Maier P, Hartmann L, Wenz F, Herskind C (2016). Cellular pathways in response to ionizing radiation and their targetability for tumor radiosensitization. Int J Mol Sci.

