# Supplementary material for: Histological response to radiotherapy is an early event in myxoid liposarcoma
Source: Virchows Arch. 2023 Aug 12;483(4):487–95. doi: 10.1007/s00428-023-03615-5 (PMC10611607; doi:10.1007/s00428-023-03615-5)
Supplement: Supplementary file 1 — Supplementary file1 (DOCX 13 KB) [file 428_2023_3615_MOESM1_ESM.docx]

**Supplementary Table 1. Details of antibodies**

| Antibody | Clone | Dilution | Source | Antigen retrieval |
| --- | --- | --- | --- | --- |
| Bcl-2 | 124 | 1:800 | Cell signalling | Tris-EDTA |
| Phospho-S6 | D57.2.2E | 1:50 | Cell signalling | Citrate |
| CAIX | polyclonal | 1:2000 | Novus | Citrate |
| Cleaved caspase-3 | polyclonal | 1:800 | Cell signalling | Citrate |
| Ki67 | D2H10 | 1:1600 | Cell signalling | Citrate |
| CD45 | 2B11 | 1:500 | DAKO | Tris-EDTA |
| CD68 | KP1 | 1:3000 | DAKO | Tris-EDTA |
| CD34 | QBEnd | 1:500 | DAKO | Tris-EDTA |
