# Supplementary figures and images for: Histological response to radiotherapy is an early event in myxoid liposarcoma
Source: Virchows Arch. 2023 Aug 12;483(4):487–95. doi: 10.1007/s00428-023-03615-5 (PMC10611607; doi:10.1007/s00428-023-03615-5)

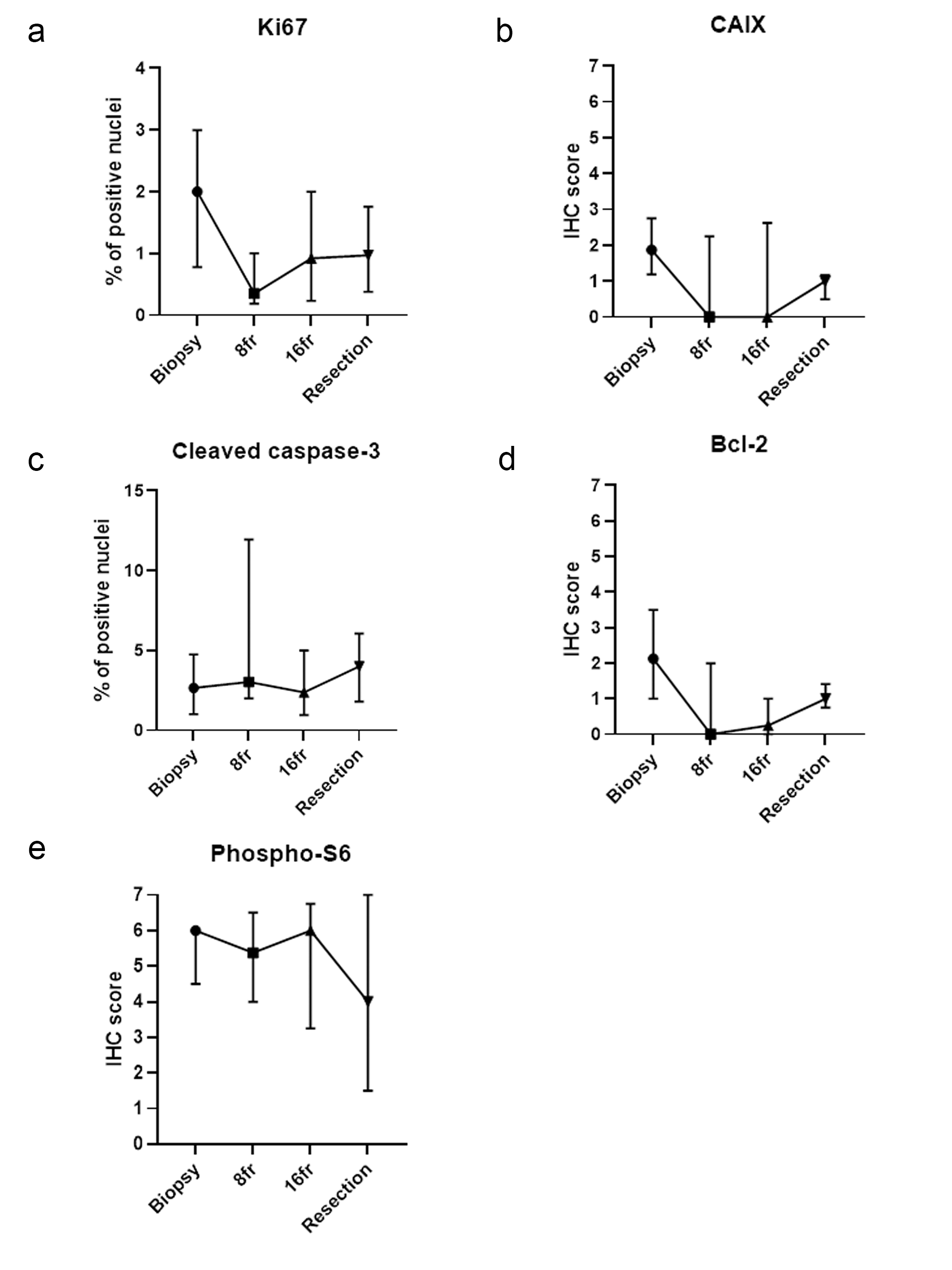

Supplement: Supplementary file 2 — Supplementary file2 (TIF 17939 KB) [file 428_2023_3615_MOESM2_ESM.tif]
